# Biomarkers for prediction of skeletal disease progression in mucopolysaccharidosis type I

**DOI:** 10.1002/jmd2.12190

**Published:** 2020-12-08

**Authors:** Troy C. Lund, Terence M. Doherty, Julie B. Eisengart, Rebecca L. Freese, Kyle D. Rudser, Ellen B. Fung, Bradley S. Miller, Klane K. White, Paul J. Orchard, Chester B. Whitley, Lynda E. Polgreen

**Affiliations:** ^1^ Department of Pediatrics University of Minnesota Minneapolis Minnesota USA; ^2^ Department of Pediatrics The Lundquist Institute at Harbor‐UCLA Medical Center Torrance California USA; ^3^ Biostatistical Design and Analysis Center, Clinical and Translational Science Institute University of Minnesota Minneapolis Minnesota USA; ^4^ School of Public Health, Division of Biostatistics University of Minnesota Minneapolis Minnesota USA; ^5^ Department of Hematology University of California, San Francisco Benioff Children's Hospital Oakland California USA; ^6^ Department of Orthopaedics and Sports Medicine Seattle Children's Hospital Seattle Washington USA

**Keywords:** biomarker, bone, cytokines, Hurler, inflammation, mucopolysaccharidosis, Scheie

## Abstract

**Background:**

Orthopedic disease progresses in mucopolysaccharidosis type I (MPS I), even with approved therapies and remains a major factor in persistent suffering and disability. Novel therapies and accurate predictors of response are needed. The primary objective of this study was to identify surrogate biomarkers of future change in orthopedic disease.

**Methods:**

As part of a 9‐year observational study of MPS I, range‐of‐motion (ROM), height, pelvic radiographs were measured annually. Biomarkers in year 1 were compared to healthy controls. Linear regression tested for associations of change in biomarkers over the first year with change in long‐term outcomes.

**Results:**

MPS I participants (N = 19) were age 5 to 16 years and on average 6.9 ± 2.9 years post treatment initiation. Healthy controls (N = 51) were age 9 to 17 years. Plasma IL‐1β, TNF‐α, osteocalcin, pyridinolines, and deoxypyridinolines were higher in MPS than controls. Within MPS, progression of hip dysplasia was present in 46% to 77%. A 1 pg/mL increase in IL‐6 was associated with −22°/year change in ROM (−28 to −15; *P* < .001), a 20 nmol/mmol creatinine/year increase in urine PYD was associated with a −0.024 Z‐score/year change in height Z‐score (−0.043 to −0.005; *P* = .016), and a 20 nmol/mmol creatinine/year increase in urine PYD was associated with a −2.0%/year change in hip dysplasia measured by Reimers migration index (−3.8 to −0.1; *P* = .037).

**Conclusions:**

Inflammatory cytokines are high in MPS I. IL‐6 and PYD were associated with progression in joint contracture, short stature, and hip dysplasia over time. Once validated, these biomarkers may prove useful for predicting response to treatment of skeletal disease in MPS I.

**SYNOPSIS**

Change in plasma and urine biomarkers over 1 year are associated with skeletal disease progression over 9 years in individuals with mucopolysaccharidosis type I.

## 1 INTRODUCTION

Mucopolysaccharidosis type I (MPS I) is a lysosomal storage disease caused by a mutation in the *IDUA* gene that manifests as central nervous system (cognitive impairment) and somatic (eg, bone, cartilage, heart, liver) abnormalities that vary broadly in severity.^1^ The severe form of MPS I, involving rapid neurocognitive deterioration and early death before 10 years of age, is designated MPS IH (for Hurler syndrome; OMIM 607014), whereas the phenotypes with less severe neurocognitive symptoms and longer lifespan are called “attenuated” (MPS IA; including the phenotypic spectrum from Hurler‐Scheie syndrome (OMIM 607015) to Scheie syndrome (OMIM 607016)). These distinct forms of MPS I are treated very differently. Children with MPS IH require hematopoietic stem cell transplantation (HSCT), sometimes supplemented with peri‐HSCT enzyme replacement therapy (ERT). It is well recognized that HSCT can alter the course of neurocognitive deterioration whereas ERT alone does not.^2^, ^3^, ^4^, ^5^, ^6^, ^7^ In contrast, children with MPS IA are treated with ERT alone which attenuates somatic disease without the inherent risks of HSCT.^8^, ^9^

HSCT and ERT therapies have considerably extended the life expectancy for patients, but significant skeletal and joint disease continues to cause severe physical disability and a negative impact on quality of life for individuals with MPS I.^3^, ^8^, ^9^, ^10^ Otherwise successfully treated patients with MPS I show one or more progressive orthopedic complications (eg, hip dysplasia, kyphosis, scoliosis, osteoarthritis) that causes chronic pain, decreased physical function, and poor quality of life.^3^, ^11^, ^12^, ^13^, ^14^ Thus, therapies to alleviate the significant debilitating disease are needed and are critical to improve the health, function and quality of life of individuals with MPS I.

Based upon animal studies, the crippling skeletal and joint disease manifestations of MPS appear to be, at least in part, inflammatory in nature.^15^, ^16^, ^17^, ^18^, ^19^, ^20^ For example, individuals with MPS I, II, and VI have high circulating levels of the inflammatory cytokine TNF‐α and these high TNF‐α levels are associated with more pain and physical disability.^12^ In animal models of MPS, elevated levels of TNF‐α, IL‐1β, and RANKL have all been implicated in MPS‐related joint disease.^16^, ^18^, ^20^

Currently, design of therapeutic clinical trials is limited by the lack of control data on the natural history of skeletal disease progression and minimal information regarding early biomarkers that predict disease progression. Without this information, the time required to demonstrate a significant clinical effect based on disease progression is protracted to the point of being impractical. Thus, identifying predictive biomarkers of disease response to treatment is critical in promoting the efficient advancement of treatments for MPS.

The goals of this prospective observational study are to measure the change in skeletal and joint disease over time in MPS I, evaluate pathways of inflammation and bone modeling and remodeling, and identify potential biomarkers that are predictive of change in bone and joint disease in MPS I. These could be tested in future clinical trials for their ability to predict MPS disease response to treatment.

## 2 MATERIALS AND METHODS

### 2.1 Study design

Plasma and urine samples from 19 participants with MPS I (14 MPS IH; 5 MPS IA), age 5 to 16 years, who were enrolled in a 9‐year, multicentered, longitudinal observational study of children and young adults with MPS (Lysosomal Disease Network and Clinicaltrials.gov NCT01521429) and had participated in the study for a minimum of 5 years were included in this analysis. Inclusion criteria were diagnosis of MPS I, age 5 to 33 years of age, and ability to travel to the study center. All participants with MPS IA were being treated with ERT for more than 1 year at study initiation. Exclusion criteria were pregnancy, participation in any other study within the past 6 months that would increase radiation exposure above 500 mrem for the calendar year, and inability to comply with study procedures.

Healthy control subjects were recruited for a separate study of the association between bone and energy metabolism.^21^ Inclusion criteria included otherwise healthy adolescents between 8 and 17 years of age. Exclusion criteria included diagnosis of diabetes mellitus, polycystic ovarian syndrome, treatment with a medication that alters insulin sensitivity, secretion, or beta‐cell mass, participation in a concurrent intervention trial, and pregnancy.

### 2.2 Biomarkers

Blood and urine samples were collected from both populations the morning after a minimum 8‐hour fast in 4 mL EDTA tubes, immediately processed, frozen, and stored at −80°C. All MPS samples were run in one batch. All control samples were run in another batch.

Markers of inflammation (interleukin‐1 β [IL‐1β], interleukin‐6 [IL‐6], tumor necrosis factor‐α [TNF‐α]), bone formation (osteocalcin [OCN]), bone resorption (deoxypyridinolines [DPD], pyridinolines [PYD]), and cartilage degradation (PYD) were measured. The bone biomarkers were chosen based on their prior use in pediatric studies of bone^22^, ^23^ and availability of well‐established assays described below. IL‐1β was measured in plasma using the Quantikine high sensitivity Human IL‐1β Immunoassay from R & D Systems (Minneapolis, MN): intra‐assay coefficient of variation (CV) was <8.2% and inter‐assay CV was <16.1%. IL‐6 was measured in plasma using the Quantikine high sensitivity Human IL‐6 Immunoassay (R & D Systems): intra‐assay CV < 6.0% and inter‐assay CV <10.5%. TNF‐α was measured in plasma using the Quantikine high sensitivity Human TNF‐α Immunoassay (R & D Systems): intra‐assay CV <6.8% and inter‐assay CV <10.7%. OCN was measured in serum using the MicroVue Osteocalcin EIA kit from Quidel Corporation (San Diego, California): intra‐assay %CV <5.0 and inter‐assay %CV <12.0. DPD was measured in urine using the MicroVue DPD EIA kit (Quidel Corporation): intra‐assay CV <8.4% and inter‐assay CV <4.8%. PYD was measured in urine using the MicroVue PYD EIA kit (Quidel Corporation): intra‐assay CV <9.9% and inter‐assay CV <11.2%.

### 2.3 Outcome measures

Shoulder flexion, elbow extension, and knee extension were measured in each joint three times and the average taken. Measurement of range‐of‐motion (ROM) was standardized at all three sites with a training manual provided for reference at each site. In addition, the same one to two individuals performed the measurements at each visit. A joint summary score was calculated as the summary of all six joints (higher number = better ROM). Height was measured with a wall‐mounted stadiometer, three times standing without shoes, and the average recorded. Each subject was repositioned between each measurement. Height Z‐score (ie, adjusted for age and sex) was calculated based on the National Center for Health Statistics 2000 data as provided by the Center for Disease Control. Tonnis angle, Reimers migration index (RMI), and Tonnis Grade were measured from anterior‐posterior pelvic radiographs by a single individual (Dr K. K. W., Pediatric Orthopedic Surgery). Tonnis angle and RMI are measurements of hip dysplasia. Tonnis Grade is a measurement of hip osteoarthritis.

### 2.4 Statistical analysis

Descriptive statistics are presented as mean ± SD for continuous variables, and as frequency and percent for nominal variables. Linear regression models were used to compare biomarkers at baseline in MPS vs controls with adjustment for BMI Z‐score for inflammatory biomarkers (TNF‐α, IL‐6, IL‐1β) and adjustment for age for bone and cartilage biomarkers (OCN, DPD, PYD). Furthermore, linear regression was used to examine the association of annual rate of change in biomarker over study years 1 to 2 with the annual rate of change in ROM over years 5 to 9 and height Z‐score over years 2 to 9 in participants with MPS, adjusted for age. Outcomes of annual rate of change for ROM and height Z‐score were calculated using linear regression to fit a slope for change in each outcome over time, per individual. Outliers of change in biomarkers were removed based on being greater than three SD from the mean and by visual inspection. Annualized rate of change of Tonnis angle and RMI were calculated for each participant and for right and left hips over study visits 7 to 9. Rather than average across hips for each participant, the hip that showed the most severe disease progression in Tonnis angle and RMI was used as the outcome in linear regression models with each of the change in biomarkers during the first year of study as independent variables. These models were further adjusted for age (months) and the first recorded value of the Tonnis angle or RMI. Statistical analysis of change in ROM, height Z‐score, Tonnis angle, and RMI over time in MPS IH vs MPS IA is not included due to low sample size within MPS groups.

Analyses and graphing were performed using R, version 3.5.3, STATA 14, Adobe Photoshop, and Prism.

## 3 RESULTS

Population characteristics are described in detail in Table 1. Of note, the study included 51 healthy controls and 19 participants with MPS I (14 MPS IH; 5 MPS IA). Controls were, on average, older than MPS participants. Controls and MPS participants were similar in BMI Z‐scores. All participants with MPS IH were treated with HSCT ≥2.9 years prior to study enrollment, and all participants with MPS IA were currently treated with ERT (laronidase). None were wheelchair dependent. Within the MPS I group, baseline patient records were examined for evidence of infection due to the potential influence on cytokine analysis; there was one participant with mild nasal congestion assumed related to seasonal allergies given normal physical exam, and one participant with tinea corporis.

**TABLE 1:** Population characteristics

	Controls	All MPS I	MPS IH	MPS IA
	N = 51	N = 19	N = 14	N = 5
Age at visit, y	14.6 ± 2.0 (9.2‐17.9)	9.7 ± 4.2 (5.0‐16.5)	8.0 ± 2.9 (5.0‐12.4)	14.3 ± 3.9 (7.4‐16.5)
Treated with HSCT, y	NA	14 (74)	14 (100)	0 (0)
Age at HSCT initiation, y	NA	1.3 ± 0.7 (0.2‐2.5)	1.3 ± 0.7 (0.2‐2.5)	NA
Treated with ERT, yes	NA	13 (68)	8 (57)^a^	5 (100)
Age at ERT initiation, y	NA	3.8 ± 4.0 (0.5‐13.3)	1.3 ± 0.6 (0.5‐2.3)	7.8 ± 4.0 (2.5‐13.3)
Time since HSCT, y	NA	6.7 ± 3.0 (2.9‐11.2)	6.7 ± 3.0 (2.9‐11.2)	NA
Time since ERT initiation, y	NA	5.5 ± 2.1 (3.1‐10.7)	4.5 ± 1.0 (3.1‐6.3)	6.9 ± 2.7 (3.6‐10.7)
Sex, female	27 (53)	8 (42)	7 (50)	1 (20)
Race				
White^b^	38 (75)	19 (100)	14 (100)	5 (100)
Black	5 (10)	0 (0)	0 (0)	0 (0)
American Indian	1 (2)	0 (0)	0 (0)	0 (0)
Mixed	7 (13)	0 (0)	0 (0)	0 (0)
Ethnicity				
Hispanic	5 (10)	1 (0.05)	0 (0)	1 (20)
Non‐Hispanic	46 (90)	18 (95)	14 (100)	4 (80)
BMI, Z‐score	1.1 ± 1.2 (−1.4 to 2.8)	0.8 ± 0.9 (−0.5 to 2.4)	0.7 ± 0.9 (−0.5 to 1.9)	1.1 ± 1.0 (0.2‐2.4)
Bone age, y	15.1 ± 2.1 (10.0‐19.0)	9.4 ± 5.0 (3.0‐19.0)	7.6 ± 3.6 (3.0‐13.5)	14.5 ± 5.0 (7.0‐19.0)
Joint Summary Score	NA	215 ± 84	249 ± 67	118 ± 41
Height Z‐score	0.3 ± 1.0 (−2.1 to 1.9)	−2.3 ± 1.1 (−4.2 to −0.7)	−2.4 ± 1.2 (−4.2 to −0.7)	−2.0 ± 0.7 (−3.2 to −1.5)
Tonnis grade (highest hip)	NA			
0		9 (50)^1^	7 (50)	2 (50)^1^
1		1 (6)	1 (7)	0
2		2 (11)	2 (14)	0
3		5 (28)	4 (29)	1 (25)
5		1 (6)	0	1 (25)
Tonnis angle right	NA	17 ± 12^(1)^ (1‐40)	18 ± 13 (1‐40)	15 ± 10^(1)^ (8‐30)
Tonnis angle left	NA	20 ± 14^(1)^ (−2 to 44)	23 ± 14 (−2 to 44)	9 ± 4^(1)^ (6‐15)
RMI right	NA	36 ± 22^(3)^ (6‐80)	36 ± 22^(2)^ (6‐80)	35 ± 22^(1)^ (15‐66)
RMI Left	NA	38 ± 18^(2)^ (11‐75)	40 ± 19^(1)^ (11‐75)	30 ± 7^(1)^ (22‐38)
IL‐1β, pg/mL	0.17 ± 0.04^(24)^	0.34 ± 0.35	0.36 ± 0.41	0.25 ± 0.05
TNF‐α, pg/mL	1.2 ± 0.4	5.7 ± 2.8	6.3 ± 3.0	4.2 ± 1.2
IL‐6, pg/mL	1.7 ± 1.3	1.9 ± 5.6^(1)^	2.3 ± 6.3	0.2 ± 0.2^(1)^
OCN, pg/mL	17.9 ± 8.3	27.1 ± 9.8	30.4 ± 9.0	17.9 ± 4.6
DPD, nmol/mmol creatinine	9.1 ± 5.0	26.0 ± 15.4	29.5 ± 15.6	16.1 ± 10.1
PYD, nmol/mmol creatinine	111.1 ± 60.1	146.2 ± 81.4	159.8 ± 80.6	108.0 ± 79.0

*Note*: Superscript numbers indicate number of missing data. Data are presented as mean ± SD (min‐max) or N (%).

Abbreviations: BMI, body mass index; DPD, urine deoxypyridinolines; ERT, enzyme replacement therapy; HSCT, hematopoietic stem cell transplantation; IL‐1β, interleukin‐1β; IL‐6, plasma interleukin‐6; OCN, plasma osteocalcin; PYD, urine pyridinolines; RMI, Reimers migration index; TNF‐α, tumor necrosis factor‐α.

^a^ Peri‐transplant ERT only.

^b^ Includes Hispanic.

**FIGURE 1:**
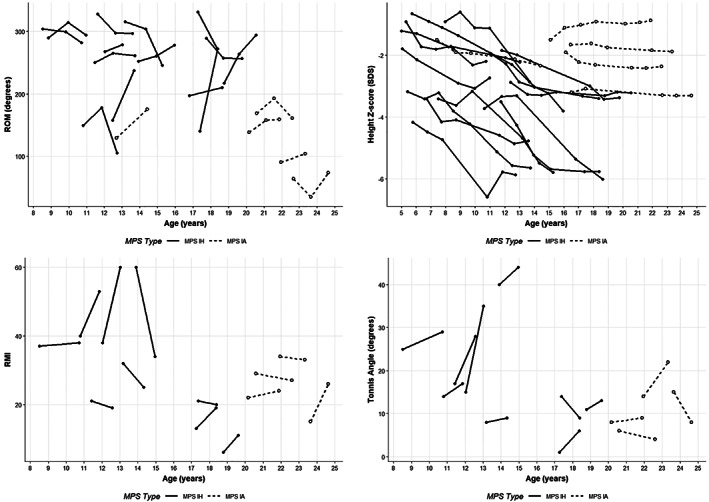
Change in joint range of motion (ROM) summary score (sum of bilateral shoulder flexion, elbow, and knee extension), height Z‐score, Reimers migration index (RMI), and Tonnis angle over time. Filled circles and solid lines = MPS IH; open circles and dotted lines = MPS IA

Growth failure and joint contractures were highly prevalent in the MPS I cohort, despite treatment with HSCT and/or ERT. All MPS IH participants and 60% with MPS IA participants had short stature by last follow up. All MPS I participants had joint contracture of at least one joint. Participants treated with only ERT (ie, MPS IA participants) compared to those treated with HSCT (ie, MPS IH participants) had more severe joint contractures based on the ROM summary score; however, they were significantly older than the HSCT treated cohort (Table 1; Figure 1). One MPS participant had undergone hip replacement due to severe osteoarthritis along with hip dysplasia, and 50% of MPS participants had some degree of hip osteoarthritis by Tonnis grade (Table 1).

Joint contracture worsened with age at entry into this observational study in the MPS IA but not in the MPS IH cohort (Figure 1). Height Z‐score decreased gradually over time in MPS IH (Figure 1). Progression of hip dysplasia was present in 46% (N = 6) by RMI and 77% (N = 10) by Tonnis angle (Figure 1).

**FIGURE 2:**
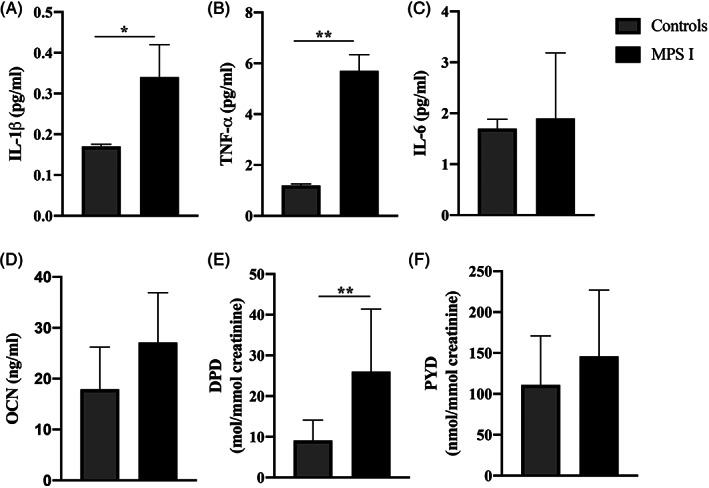
Inflammatory, A‐C, and bone, D‐F, biomarker concentrations in mucopolysaccharidosis type I (MPS I) compared to healthy controls. Mean ± SEM is shown. *P* ‐ values adjusted for BMI Z‐score, A‐C, or age, D‐F. *P < .05; **P < .001. DPD, urine deoxypyridinolines; IL‐1β, interleukin‐1β; IL‐6, plasma interleukin‐6; OCN, plasma osteocalcin; PYD, urine pyridinolines; TNF‐α, tumor necrosis factor‐α

TNF‐α, IL‐1β, and DPD were higher in MPS vs controls (all *P*‐values <.05), and there was no significant difference in IL‐6, OCN, or PYD values between groups (Figure 2). Regression analysis in MPS I revealed that the within‐individual change in IL‐6 over the first 2 years of the study was significantly associated with the change in joint ROM over years 5 to 9 (−22°/year change in ROM for every 1 pg/mL increase in IL‐6 (95%CI −29 to −15; *P* < .001); this association remained significant when repeating the analysis with only MPS IH participants (*P* < .001) confirming that the association is not due to differences between MPS IH and MPS IA.

There was also a within‐individual change of −0.024 SDS/year in height Z‐score for every 20 nmol/mmol creatinine/year change in urine PYD (95% CI −0.043 to −0.005; *P* = .016) and a −2.0°/year in RMI for every 20 nmol/mmol creatinine/year change in urine PYD (95% CI −3.8 to −0.1; *P* = 0.037). There were no other statistically significant associations between biomarkers and joint ROM, height Z‐score, RMI, or Tonnis angle (Table 2).

**TABLE 2:** Associations between change in biomarkers over first year of study with annualized change in joint ROM over years 5 to 9, annualized height Z‐score over years 2 to 9, annualized Tonnis angle, and RMI over years 7 to 9

		Estimated annual rate of change (95% CI)	*P*‐value
Joint ROM over years 5‐9	IL‐1β (per 0.1 pg/mL)	−11.0 (−30.6, 8.5)	.246
	TNF‐α (per 10 pg/mL)	−43.7 (−129.8, 42.5)	.295
	IL‐6 (per 1 pg/mL)	−22.0 (−28.7, −15.3)	<.001
	OCN (per 10 pg/mL)	4.1 (−12.7, 20.8)	.614
	DPD (per 20 nmol/mmol)	18.2 (−22.9, 59.2)	.358
	PYD (per 20 nmol/mmol)	0.4 (−4.5, 5.4)	.857
Height Z‐score over years 2‐9	IL‐1β (per 0.1 pg/mL)	−0.005 (−0.10, 0.089)	.908
	TNF‐α (per 10 pg/mL)	−0.06 (−0.48, 0.35)	.746
	IL‐6 (per 1 pg/mL)	−0.03 (−0.10, 0.04)	.365
	OCN (per 10 pg/mL)	−0.04 (−0.12, 0.04)	.291
	DPD (per 20 nmol/mmol)	−0.01 (−0.16, 0.14)	.849
	PYD (per 20 nmol/mmol)	−0.024 (−0.043, −0.005)	.016
Tonnis angle over years 7‐9	IL‐1β (per 0.1 pg/mL)	−0.91 (−5.10, 3.27)	.634
	TNF‐α (per 10 pg/mL)	−5.03 (−23.61, 13.55)	.550
	IL‐6 (per 1 pg/mL)	0.08 (−0.70, 0.86)	.826
	OCN (per 10 pg/mL)	−0.92 (−5.54, 3.69)	.662
	DPD (per 20 nmol/mmol)	−5.22 (−16.14, 5.71)	.303
	PYD (per 20 nmol/mmol)	0.28 (−0.79, 1.36)	.564
RMI over years 7‐9	IL‐1β (per 0.1 pg/mL)	−0.01 (−9.33, 9.31)	.998
	TNF‐α (per 10 pg/mL)	11.47 (−23.12, 46.06)	.467
	IL‐6 (per 1 pg/mL)	−0.75 (−2.27, 0.76)	.286
	OCN (per 10 pg/mL)	1.22 (−8.03, 10.47)	.772
	DPD (per 20 nmol/mmol)	−8.11 (−30.03, 13.81)	.418
	PYD (per 20 nmol/mmol)	−1.99 (−3.84, −0.14)	.037

*Note*: All estimates adjusted for age in months and the first recorded value of Tonnis angle or RMI for these measures.

Abbreviations: DPD, urine deoxypyridinolines; IL‐1β, interleukin‐1β; IL‐6, plasma interleukin‐6; OCN, plasma osteocalcin; PYD, urine pyridinolines; RMI, Reimers migration index; ROM, range of motion summary score (sum of bilateral shoulder flexion, elbow, and knee extension); TNF‐α, tumor necrosis factor‐α.

## 4 DISCUSSION

In this study, we report several important findings. First, although the presence of inflammation has been found in animal models of MPS I and treatment naïve patients with MPS IH,^24^, ^25^ our study identified elevated levels of biomarkers of inflammation in treated MPS I individuals compared to healthy controls. Second, we present data showing that despite current therapies joint contractures, short stature, hip dysplasia, and osteoarthritis persists, and may even worsen over time. Third, we identified PYD as a prognostic biomarker of future growth and hip dysplasia and IL‐6 of future rate of change in joint ROM. Given that joint contractures and skeletal disease progression are among the most significant, impactful contributors to persistent suffering and disability in individuals with MPS I, these findings of a link between biomarker change and future change in a clinical outcome have important implications for understanding disease pathology and potentially predicting therapeutic impact in future clinical trials.

Biomarkers obtained prior to the onset of disease that are prognostic of the disease‐related severity or predictive of treatment response are used in other, non‐MPS, populations. In the skeletal system, biomarkers of bone turnover are prognostic of the risk of fracture in adult populations^26^, ^27^, ^28^ and osteoporosis treatment efficacy in bisphosphonate clinical trials.^29^, ^30^, ^31^ The predictability of inflammatory and cartilage turnover markers for disease response to treatment has been evaluated in individuals with inflammatory joint disease treated with TNF‐α inhibitors as well. These studies have found associations between baseline C‐reactive protein, TNF‐α, or markers of cartilage turnover and response to treatment.^32^ Additional biomarkers of inflammation and cartilage turnover (eg, cartilage oligomeric matrix protein, soluble E‐selectin, and intercellular adhesion molecule‐1) decrease with treatment and have variable predictability of disease response to therapy in other inflammatory skeletal diseases such as juvenile idiopathic arthritis, rheumatoid arthritis, and ankylosing spondylitis.^32^, ^33^, ^34^ Similar inflammatory and cartilage biomarkers may be useful in predicting disease response to treatments over time in individuals with MPS.

In MPS, prior studies have shown a change in a biomarker in response to therapy, but not an association with future change in bone or cartilage outcomes. One study found that heparan sulfate (HS) and dermatan sulfate (DS), two glycosaminoglycans (GAGs) that accumulate in MPS I, decrease after initiation of ERT, but HS or DS did not correlate with clinical outcomes in patients with MPS I treated with ERT.^35^ Another potential biomarker is heparin cofactor II‐thrombin complex, which also decreases in response to treatment with ERT and HSCT, and increases with the clinical response of enzyme infusion reaction of flushing.^36^, ^37^ However, we know of no studies attempting to use these markers to predict *future* bone or cartilage disease using clinical outcomes.

We found significant systemic inflammation in MPS I, demonstrated by the elevated IL‐1β and TNF‐α cytokine levels compared to healthy controls. Elevated IL‐1β cytokine levels are synonymous with NLRP3 inflammasome activation.^38^ Our findings are consistent with preclinical reports of the importance of TNF‐α and inflammasome activation in MPS disorders. CNS and somatic disease in MPS animal models have been shown to be driven, in part, by the GAG‐induced inflammatory response through the TLR4/MyD88 signaling pathways, resulting in increased secretion of TNF‐α from cells such as microglia and macrophage, along with NLRP3 inflammasome activation.^15^, ^16^, ^17^, ^18^, ^39^, ^40^, ^41^, ^42^, ^43^, ^44^, ^45^, ^46^, ^47^, ^48^, ^49^, ^50^, ^51^ This may be most consistent with the elevated IL‐1β and TNF‐α levels we found in children and adolescents with MPS I, but at present, this is speculative.

Surprisingly, although we found an association between PYD and growth, we did not find a similar association for DPD, a similar biomarker of bone and cartilage turnover, and growth. We speculate that this is because PYD originates from both bone and cartilage, but DPD is from only bone,^52^, ^53^ and would suggest that chondrocyte abnormalities leading to abnormal endochondral growth and increased cartilage turnover, more so than abnormal bone modeling/remodeling, play a significant role in the development of short stature in children with MPS I.

At first glance, the fact that there was not a statistically significant within‐individual change in ROM over time in our MPS I cohort may seem to detract from our findings of a significant association IL‐6 with ROM summary score. However, we believe the lack of statistically significant within‐individual change in ROM summary score may be due to the relative short duration of measurement, small sample size, and the variability of measurement inherent in the ROM outcome. Furthermore, the estimated change in ROM (−22°/y) that is associated with a 1 pg/mL change in IL‐6 is far greater than what would be considered clinically significantly. This, along with the lack of associations between IL‐1β and TNF‐α cytokine levels with joint contracture changes over time, may also simply be due to the same limitations which are common problems with studies of rare diseases.

A variety of factors can influence bone and inflammatory biomarkers. Our samples were collected in the morning after a minimum 8‐hours fast to avoid variability related to hormonal changes throughout the day and diet. Although inflammatory biomarkers are increased in adolescents with obesity,^54^ we have previously shown that obesity does not influence levels of the bone biomarkers osteocalcin and bone specific alkaline phosphatase in MPS I.^21^ However, bone biomarkers do change with age and pubertal stage.^55^ For this reason, we adjusted our analyses using bone biomarkers for age but not BMI Z‐score. Inflammatory biomarkers are not influenced by age in our cohort of individuals with MPS I (data not shown), but as already mentioned, they are influenced by obesity. Thus, we included BMI Z‐score in the analysis of the inflammatory biomarkers.

This study is limited by the difference in mean age between our MPS and control cohorts, as well as a small sample of MPS IA participants making comparisons between the MPS I groups not statistically feasible. To address this difference in age between groups, we adjusted for age in our analyses of bone biomarkers which are known to change with age and pubertal stage.

## 5 CONCLUSIONS

In conclusion, inflammasome disease is a prominent feature, or perhaps the driving pathology, of skeletal system disease as demonstrated by increased levels of cytokines and is a possible therapeutic target for treatments of MPS I in addition to HSCT and ERT. Biomarkers that are associated with long‐term clinical outcomes, such as we report here, are needed to most effectively and efficiently test potential therapies due to the rarity of the disease and relatively slow rate of disease progression. We have identified two biomarkers that were prognostic of long‐term change in growth, joint contractures, and hip dysplasia. Once validated as predictive of long‐term response to a therapy aimed at treating the joint and skeletal manifestations of MPS I, these biomarkers could be used for predicting outcomes in both clinical practice and clinical trials.

## CONFLICT OF INTERESTS

Dr T. C. L. receives research support from Sanofi‐Genzyme. Dr J. B. E. has received honoraria, consulting fees, and/or research support from ArmaGen, JCR pharmaceuticals, Orchard Therapeutics, Bluebird Bio, Shire/Takeda, and Sanofi‐Genzyme. Ms R. L. F. declares that she has no conflict of interest. Dr K. D. R. declares that he has no conflict of interest. Dr E. B. F. is a consultant for BioMarin Pharmaceuticals and Ascendis. Dr B. S. M. is a consultant for AbbVie, Ascendis, Ferring, Novo Nordisk, Pfizer, Sandoz, and Versartis and has received research support from Alexion, Ascendis, Endo Pharmaceuticals, Genentech, Genzyme, Novo Nordisk, Opko, Sandoz, Sangamo, Shire, Tolmar, and Versartis. Dr K. K. W. is a consultant for BioMarin Pharmaceuticals, has received honoraria from BioMarin Pharmaceuticals, Sanofi‐Genzyme, has grant funding from BioMarin Pharmaceuticals and Ultragenyx, and receives royalties from UpToDate.com. Dr C. B. W. is a consultant for Sanofi‐Genzyme. Dr P. J. O. receives research and clinical trial support from Sanofi‐Genzyme, Horizon, Magenta, and Bluebird Bio. Dr L. E. P. is a speaker and consultant for Sanofi‐Genzyme, has received research support from Sanofi‐Genzyme, Shire, BioMarin, and Pfizer‐Therachon, and is a consultant for Sangamo, Immusoft, Pfizer‐Therachon, and BioMarin.

## AUTHOR CONTRIBUTIONS

**Troy C. Lund** and **Terence M. Doherty**: Wrote the first draft of the manuscript and are thus co‐first authors. **Rebecca L. Freese** and **Kyle D. Rudser**: Performed statistical analysis. **Ellen B. Fung**, **Bradley S. Miller**, **Chester B. Whitley**, and **Lynda E. Polgreen**: Designed the research studies. **Ellen B. Fung**, **Bradley S. Miller**, and **Lynda E. Polgreen**: Conducted experiments and acquired data. **Klane K. White**: Interpreted radiographs. Finally, all authors provided critical feedback and helped shape the analysis and manuscript.

## ETHICS APPROVAL AND PATIENT CONSENT

All procedures followed were in accordance with the ethical standards of the responsible committee on human experimentation (institutional and national) and with the Helsinki Declaration of 1975, as revised in 2000 (5). Informed consent was obtained from all patients for being included in the study.
